# Self-reported infertility diagnoses and treatment history approximately 20 years after fertility treatment initiation

**DOI:** 10.1186/s40738-021-00099-2

**Published:** 2021-03-12

**Authors:** Alesia M. Jung, Stacey A. Missmer, Daniel W. Cramer, Elizabeth S. Ginsburg, Kathryn L. Terry, Allison F. Vitonis, Leslie V. Farland

**Affiliations:** 1 Mel and Enid Zuckerman College of Public Health, University of Arizona, 1295 N. Martin Avenue, Tucson, AZ 85724 USA; 2 Department of Obstetrics, Gynecology, and Reproductive Biology, College of Human Medicine, Michigan State University, East Lansing, MI USA; 3 Department of Epidemiology, Harvard T.H. Chan School of Public Health, Boston, MA USA; 4 Department of Obstetrics and Gynecology, Brigham and Women’s Hospital and Harvard Medical School, Boston, MA USA

**Keywords:** Assisted reproductive technology; InVitro fertilization, Fertility treatment; infertility; validity; reliability, Epidemiology, Fertility, Endometriosis, Polycystic ovary syndrome

## Abstract

**Background:**

Infertility history may have important implications for clinical practice and scientific discovery. Previous research on the validity of self-reported infertility measurements has been limited in scope and duration (< 5 years). In this study, we validated self-reported infertility history measures 15–23 years after fertility treatment initiation among women who utilized assisted reproductive technology (ART).

**Methods:**

Women who received ART treatments from three Boston infertility clinics and who enrolled in a prior study (1994–2003) were re-contacted in 2018 for the AfteR Treatment Follow-up Study (ART-FS). Infertility history was collected from clinical records and two self-report questionnaires (at ART initiation and at ART-FS enrollment). Treatment history included specific details (fresh or frozen embryo transfers, number of cycles) and treatment recall prior to ART initiation. Self-reported infertility diagnoses included polycystic ovary syndrome (PCOS), endometriosis, uterine factor infertility, tubal factor infertility, diminished ovarian reserve/advanced maternal age, male factor infertility, and other/unknown. We compared self-reported measures from 2018 to self-reported and clinical data from prior study initiation, using Cohen’s kappa, sensitivity, specificity, and 95% confidence intervals.

**Results:**

Of 2644 women we attempted to recontact, 808 completed the ART-FS, with an average follow-up of 19.6 years (standard deviation: 2.7). Recall of fertility treatment usage had moderate sensitivity (IVF = 0.85, Clomiphene/Gonadotropin = 0.81) but low specificity across different infertility treatment modalities (IVF = 0.63, Clomiphene/Gonadotropin = 0.55). Specific IVF details had low to moderate validity and reliability with clinical records. Reliability of recalled infertility diagnosis was higher when compared to self-report at ART initiation (PCOS K = 0.66, Endometriosis K = 0.76, Tubal K = 0.73) than when compared to clinical records (PCOS K = 0.31, Endometriosis K = 0.48, Tubal K = 0.62) and varied by diagnosis.

**Conclusions:**

The ability of women to recall specific IVF treatment details was moderately accurate and recall of self-reported infertility diagnosis varied by diagnosis and measurement method.

**Supplementary Information:**

The online version contains supplementary material available at 10.1186/s40738-021-00099-2.

## Background

Infertility affects approximately 10–15% of couples in the United States [1]. Utilization of infertility treatments, such as assisted reproductive technology (ART), have increased in the past three decades [2–4]. As ART usage increases, so does interest in understanding how women’s infertility and treatment history affect long-term health outcomes. Previous research suggests that women who experience infertility, subfertility, or reduced parity and women who utilize fertility treatments may have increased risk of certain chronic diseases [5–10]. To assess infertility history in epidemiologic studies, accurate and feasible measures of infertility and fertility treatment history are required.

A recent systematic review of ART-based validation studies indicated a lack of rigorous publications on the validation of routinely collected data from fertility populations [11]. While medical records are often the “gold standard” to collect information, utilizing medical records may not always be feasible, particularly for epidemiologic studies that have a large sample size or are population-based. Moreover, information on lifestyle factors (e.g. smoking history, diet, physical activity) that may serve as potential confounding [12] or mediating variables [13] may be absent from medical records, inconsistently documented, or inaccurately recalled. Self-reported measures are widely utilized in epidemiologic research and are often considered more cost-effective. However, there is insufficient research on the accuracy of self-reported measures of infertility, especially over an extended period of follow-up. Understanding recall after an extended follow-up period is especially important for research related to chronic health conditions that may have a significant lag between exposure and disease onset. Prior research on the validity of recalled infertility history and fertility treatment has been limited in duration of follow-up, with prior studies ranging from several months to a few years [14–19]. In research that followed some participants for a longer duration of time (maximum 17 years), only a minority of participants (< 20%) were followed for 8 or more years [20]. To overcome these previous limitations, our study evaluated women’s recall of infertility and treatment history approximately 20 years after treatment initiation and compared self-reported measures captured in 2018 to medical records and self-reported data collected at prior study initiation (1994–2003).

## Methods

The details of recruitment and participation in the original IVF Study (IVF study) have been described previously [21, 22]. Briefly, from 1994–2003 and 1999–2003, 2688 couples newly enrolled in in vitro fertilization (IVF) treatments were recruited from three IVF clinics near Boston, Massachusetts. At enrollment, medical history and lifestyle factors were obtained via a self-administered questionnaire prior to treatment. IVF treatment and outcome data were abstracted for up to six cycles from clinical records. This study was approved by the Institutional Review Board of Brigham and Women’s Hospital in Boston, Massachusetts.

In 2018, 15–23 years after enrollment in the IVF study, women were recontacted and asked to participate in the AfteR Treatment Follow-up Study (ART-FS). An initial recontact letter was mailed to women using her most recent address in the Mass General Brigham (formerly Partners Health Care) electronic health record system, used by two of the largest healthcare providers in Massachusetts. If no address was available, the address from the IVF Study record was used. Study data were collected and managed using Research Electronic Data Capture (REDCap) tools hosted by Brigham and Women’s Hospital [23, 24]. REDCap is a HIPAA-compliant, secure, web-based software platform designed to support data capture for research studies. Women were directed to use a provided REDCap study link to complete the survey online. Participants had the option to return a pre-paid postcard to request a paper copy of the questionnaire. If women did not reply to the initial letter, an additional letter was subsequently distributed 2–3 weeks later. If either the initial or subsequent letter was returned due to an incorrect address, we searched for participant’s addresses using an online search engine (https://premium.whitepages.com/, accessed April – June 2018) using exact matches to names and birth dates. Recontacted women were eligible to participate in the ART-FS. Those who completed the questionnaire, constituting consent, were included in analyses.

### Data collection

#### Medical history and lifestyle factors

Medical history and lifestyle factors were obtained from self-administered questionnaires collected between 1994–2003 during the IVF Study. Information on a variety of domains including age, race/ethnicity, religion, marital status, highest level of completed education, cigarette smoking history, depression history, reproductive history, gravidity, occupational and environmental exposures, and previous pregnancy outcomes (therapeutic abortion, miscarriage or stillbirth, ectopic (tubal) pregnancy, liveborn pregnancy, molar pregnancy) were collected.

#### Fertility treatment history

**Fig. 1:**
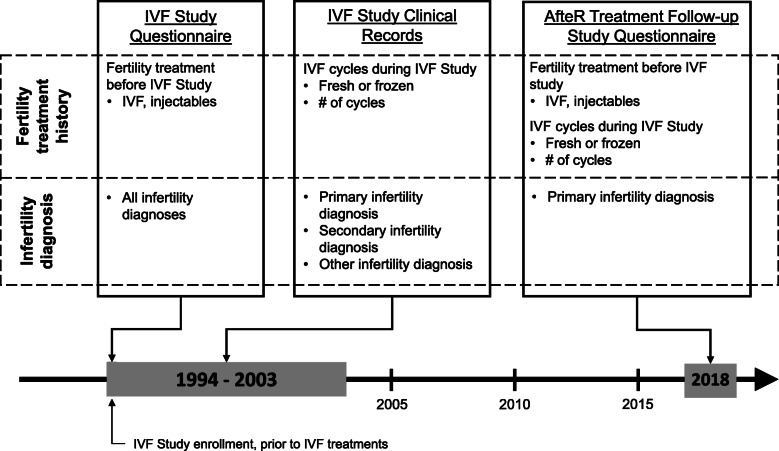
Description of data sources from AfteR Treatment Follow-up Study and IVF Study

Information on fertility treatment history was collected from three sources: i) the IVF Study clinical records, ii) the IVF Study self-reported questionnaire, and iii) the ART-FS questionnaire (Fig. 1). We compared treatment recall across two periods of time: i) prior to the IVF Study enrollment and ii) during the IVF Study. The IVF Study questionnaire was completed during study enrollment, prior to start of IVF treatments. The questionnaire asked about ever use of fertility treatments prior to IVF Study enrollment. Women were asked: “Have you previously received IVF or GIFT?” and “Have you previously received clomid or pergonal to stimulate your ovaries?” To collect information on fertility treatment history *during* IVF Study enrollment, we utilized clinical records on the number of cycles of fresh or frozen embryo transfer IVF each woman received.

On the ART-FS questionnaire, women were asked about their treatment across three time points: i) prior to IVF Study enrollment, ii) during the IVF Study, and iii) after the IVF Study. Time periods i) and ii) are compared in this analysis. Women were asked, “How many cycles of the following types of fertility treatments did you undergo before you began the [IVF Study] in [start month and year]?” with the following response options: Clomid, Gonadotropin injections, fresh embryo transfer IVF, and frozen embryo transfer IVF (range from 0 to 7+ cycles). Women were also asked to recall their fertility treatment *during* the IVF Study (“How many cycles of the following types of fertility treatments did you undergo between [start of IVF Study participation month and year] and [end of IVF Study participation month and year]?”). Women could report the number of IVF cycles separately for fresh and frozen embryo transfers (range 0 to 7+).

#### Infertility diagnoses

In the IVF Study, infertility diagnoses were collected from two sources: i) clinical records and ii) self-reported questionnaires. On the IVF Study questionnaire, women were asked: “What is your understanding of the cause(s) of your fertility problem?” and could self-report (Yes or No) multiple infertility problems: blocked or absent tubes, cervical problems, Diethylstilbestrol exposure, a double or divided uterus, endometriosis, male factor (low sperm count, etc), fibroids, polycystic ovaries, and other with no indication of priority (primary, secondary, etc). Given the structure of the questionnaire, infertility problems with missing responses were assumed to indicate the absence of that condition if at least one other infertility problem was indicated. On the IVF Study questionnaire, a woman reporting “fibroids” was categorized as having “Uterine factor infertility” and “blocked or absent tubes” was categorized as having “Tubal factor infertility”. If she reported a write-in response that included “perimenopausal”, “age” or “premature ovarian failure” she was categorized as having “Diminished ovarian reserve/Increased maternal age”. In clinical records, codes belonging to diagnostic groups were reviewed and categorized to align with the infertility diagnosis categories that were defined for the analysis (PCOS, Endometriosis, Uterine factor infertility, Tubal factor infertility, Diminished ovarian reserve/Increased maternal age, Male factor infertility, Other/unknown).

On the ART-FS questionnaire, women were asked “What do you remember as being the primary reason for why you utilized infertility treatments in the IVF Study starting in [start month and year]?” Women reported their primary infertility diagnosis (PCOS, endometriosis, uterine factor infertility, tubal factor infertility, diminished ovarian reserve, male factor infertility, increased maternal age, or other). Responses of other or missing responses were categorized as Other/Unknown.

### Statistical analysis

To assess participant differences by participation in the ART-FS, we compared women who enrolled in the ART-FS to those who did not enroll. Specifically, we assessed differences in medical and lifestyle factors reported at enrollment and clinical outcomes from the IVF Study. To evaluate the accuracy of self-reported treatment history, we calculated the validity and reliability of treatment history reported at ART-FS compared to report on IVF Study questionnaire. We looked at use of IVF, Clomiphene or Gonadotropin injections, and any fertility treatment, considering the IVF Study self-report as the gold standard. We also calculated validity and reliability of self-reported IVF treatment details from the ART-FS compared to IVF Study medical records. Usage of fresh cycles and frozen cycles (yes or no) were compared. Similarly, accuracy of number of IVF cycles (fresh, frozen, and fresh and frozen combined) was evaluated.

To evaluate recall of infertility diagnosis, we compared self-reported primary infertility diagnosis from the ART-FS to self-reported diagnoses from the IVF Study. Women could self-report multiple infertility diagnoses at IVF study enrollment, but only a primary infertility diagnosis at ART-FS. Therefore, we considered two groups: i) a restricted sample of women who self-reported one infertility diagnosis and ii) a sample of all women who reported any number of diagnoses during the original IVF Study, where “valid recall” was classified as one of the diagnoses reported during the IVF Study was recalled as the primary infertility diagnosis on ART-FS. We also compared self-reported primary infertility diagnosis from the ART-FS to i) the primary clinical diagnosis only and ii) any clinical diagnosis (primary, secondary or other), abstracted from clinical records, when one of the clinical diagnoses recorded during original IVF Study was recalled as the primary infertility diagnosis on ART-FS we classified this as “valid recall”.

For all analyses, reliability was calculated as either Cohen’s kappa coefficient (K, a measure of inter-rater agreement for binary items) or weighted Cohen’s kappa coefficient (K_w_, for inter-rater agreement of items with more than two categories). Kappa coefficients take into consideration the possibility of agreement between raters occurring by chance, so they are thought to be more robust than percent agreement (another measure of inter-rater reliability), though more conservative [25]. The kappa coefficient is widely used in agreement studies of categorical data though it has been noted to be vulnerable to the prevalence of the underlying disease and the tendencies of raters to classify test results a certain way [26]. The kappa coefficient has been used previously in studies examining recall. Some examples include the recall of menstrual irregularity [27], recall of health care resource utilization compared to abstracted medical records [28], and recall of medication use compared to prescription database records [29]. Validity was calculated as sensitivity and specificity. 95% confidence intervals (95% CIs) were calculated for all measures. Statistical analyses were conducted using SAS v9.4 software (Cary, NC).

In sensitivity analyses, we stratified the study population by those who reported receiving additional IVF treatments after the IVF Study and repeated our analyses comparing accuracy of self-reported treatment history at ART-FS to treatment history from the IVF Study questionnaire and clinical records to see if their recall differed from those who did not have additional IVF treatments. We also considered the possibility that women in the IVF Study might have received further infertility diagnosis information during additional clinical treatments, which could affect their recall during the ART-FS. We repeated our main analyses comparing primary infertility diagnosis reported during the ART-FS to self-reported diagnoses from IVF Study enrollment and diagnoses from IVF Study clinical records under two scenarios: (i) excluding women who received additional IVF treatments after their participation in the IVF Study and (ii) excluding women who received more than two IVF cycle treatments during the IVF Study.

## Results

**Fig. 2:**
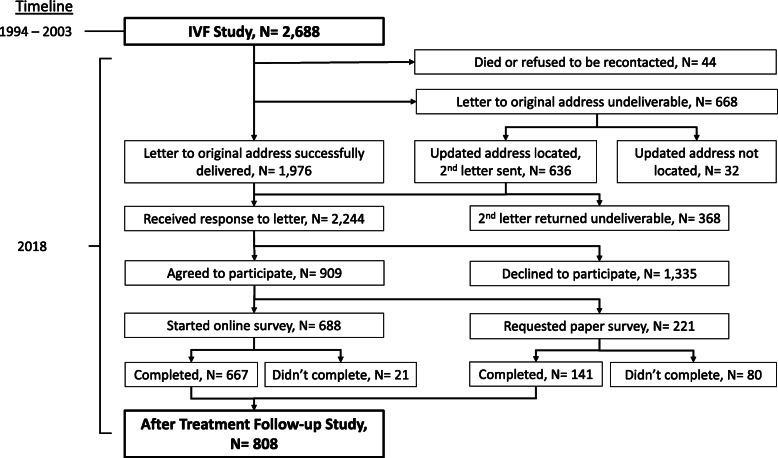
AfteR Treatment Follow-up Study participants recontacted and recruited from the IVF Study

Of the 2644 women in the IVF Study, 2244 (85%) were successfully recontacted and 909 consented (41% of those recontacted, Fig. 2). Of these women, 808 women (89%) completed the ART-FS questionnaire and were included in the analyses. Women who completed the ART-FS (completers) among those successfully recontacted had on average 19.6 years (standard deviation (SD) 2.7) between treatment initiation and follow-up. Completers were more likely to be non-Hispanic white, have completed graduate school, and were more frequently never smokers at the time of enrollment in the IVF Study, compared to those who did not complete the ART-FS (non-completers) (Table 1). We saw no meaningful difference in age, marital status, use of depression medication, and history of pregnancy at and history of miscarriage reported at IVF Study enrollment between completers and non-completers. Completers were more likely to have had at least one successful IVF cycle (resulted in a livebirth or at least a chemical pregnancy with unknown pregnancy outcome) during the IVF Study than non-completers. According to clinical records, 98.6% of our study sample had at least one fresh IVF cycle and 20.2% had at least one frozen IVF cycle between their enrollment and end of follow-up in the IVF Study (Table 1).

**Table 1:** Demographics of women in IVF Study (1994–2003), by response to ART-FS (2018), *N* = 2644

	ART-FS status		
	Recontacted and completed	Recontacted and did not complete/consent	Not successfully recontacted
	N (%)	N (%)	N (%)
	808	317	1519
**At enrollment of IVF Study**			
Year of first IVF cycle of IVF Study			
1994–1998	391 (48.4)	150 (47.3)	693 (45.6)
1999–2003	417 (51.6)	167 (52.7)	826 (54.4)
Age (years) at first cycle, mean (SD)	35.4 (4.0)	36.8 (4.2)	35.0 (4.4)
Race/Ethnicity			
Non-Hispanic white	759 (93.4)	282 (89.0)	1330 (87.9)
Black	12 (1.5)	12 (3.8)	63 (4.2)
Hispanic	10 (1.2)	8 (2.5)	27 (1.8)
Asian	22 (2.7)	11 (3.5)	81 (5.4)
Other	4 (0.5)	4 (1)	12 (0.8)
Religion			
Roman Catholic	400 (49.5)	170 (54.0)	790 (52.2)
Protestant	203 (25.1)	76 (24.1)	341 (22.5)
Jewish	91 (11.3)	26 (8.3)	116 (7.7)
Other	27 (3.3)	8 (2.5)	87 (5.8)
No organized religion	84 (10.4)	35 (11.1)	179 (11.8)
Marital status			
Married	793 (98.1)	307 (96.9)	1485 (97.9)
Not married	15 (1.9)	10 (3.2)	32 (2.1)
Highest education completed			
Less than college, including technical schools	112 (13.9)	76 (24.0)	401 (26.5)
College	359 (44.4)	133 (42.0)	647 (42.7)
Graduate school	336 (41.6)	108 (34.1)	468 (30.9)
Cigarette smoking status			
Current smoker	34 (4.2)	33 (10.4)	112 (7.4)
Former smoker	209 (25.9)	104 (32.9)	446 (29.4)
Never smoker	564 (69.8)	179 (56.7)	957 (63.2)
Ever had depression requiring medication			
Yes	73 (9.1)	27 (8.6)	141 (9.3)
No	731 (90.9)	287 (91.4)	1372 (90.7)
Gravidity (have ever been pregnant)			
Nulligravid	408 (50.7)	145 (46.0)	719 (47.4)
Gravid	397 (49.3)	170 (54.0)	799 (52.6)
Parous (had at least one prior viable pregnancy)	191 (23.9)	84 (27.1)	349 (23.2)
History of miscarriage or stillbirth	181 (22.6)	73 (23.6)	370 (24.6)
**IVF Study Clinical Records**			
Mean time between treatment initiation and 2018 follow-up (yrs), (SD)	19.6 (2.7)	19.5 (2.8)	19.5 (2.7)
Mean IVF cycles (SD)	2.16 (1.3)	2.15 (1.3)	2.16 (1.3)
Total IVF cycles resulting in transfer			
0	25 (3.1)	5 (1.6)	31 (2.0)
1	354 (43.8)	145 (45.7)	694 (45.7)
2	236 (29.2)	84 (26.5)	402 (26.5)
3	103 (12.8)	56 (17.7)	233 (15.3)
4+	90 (11.1)	27 (8.5)	159 (10.5)
Cancelled IVF cycles			
1	130 (16.1)	72 (22.7)	264 (17.4)
2	29 (3.6)	17 (5.4)	70 (4.6)
3+	7 (0.7)	4 (1.3)	20 (1.3)
Had at least one successful IVF cycle^a^	444 (55.0)	99 (31.2)	699 (46.0)
Had at least one non-successful IVF cycle^b^	483 (56.6)	209 (65.9)	924 (60.8)
Cycle with pregnancy loss^b^			
1	148 (18.3)	41 (12.9)	283 (18.6)
2+	22 (2.7)	17 (5.4)	52 (3.4)
Cycle with non-successful non-loss pregnancy outcome ^b^			
1	221 (27.4)	102	410
2	123 (15.2)	44	240
3	43 (5.3)	25	81
4+	21 (2.6)	11	51
Primary infertility diagnosis (clinical records)			
Polycystic ovarian syndrome	41 (5.1)	6 (2.0)	83 (5.5)
Endometriosis	111 (13.7)	32 (10.1)	202 (13.3)
Uterine factor infertility	18 (2.2)	11 (3.5)	42 (2.8)
Tubal factor infertility	133 (16.5)	73 (23.0)	326 (21.5)
Diminished ovarian reserve/Increased maternal age	56 (6.9)	29 (9.2)	112 (7.4)
Male factor infertility	279 (34.5)	90 (28.4)	490 (32.3)
Other/Unknown	162 (20.1)	67 (21.1)	253 (16.7)
Missing	8 (1.0)	9 (2.8)	11 (0.7)

*ART-FS* AfteR Treatment Follow-up Study, *IVF* in vitro fertilization, *SD* standard deviation

Column totals may not sum to 100% because individuals with missing responses have not be included

^a^ Successful IVF cycle included participant having a livebirth or participant having at least a chemical pregnancy, but pregnancy outcome is not known

^b^ Non-successful non-loss pregnancy outcomes included: never became pregnant and therapeutic abortion. Pregnancy loss outcomes included: participant having a chemical pregnancy (that didn’t result in livebirth), molar pregnancy, ectopic pregnancy, spontaneous abortion, and stillbirth

When we evaluated the reliability between self-reported fertility treatment prior to the IVF Study reported during the IVF Study and during the ART-FS, sensitivity and specificity values were consistent across different fertility treatment modalities (prior use of IVF: sensitivity = 0.85, specificity = 0.63; prior use of Clomiphene or Gonadotropin injections: sensitivity =0.81, specificity = 0.55; prior use of any fertility treatment: sensitivity = 0.85, specificity = 0.52) (Table 2). We also compared recall of specific IVF treatment details (type of transfer, number of cycles), comparing self-reported data from the ART-FS to clinical records. Sensitivity of recall of ever use of fresh IVF cycles was high (0.88, 95% CI 0.86, 0.90) but specificity was low (0.27, 95% CI 0.01, 0.54) (Table 3). For frozen cycles, sensitivity was 0.56 (95% CI 0.49, 0.64) and specificity was 0.71 (95% CI 0.68, 0.75). K_w_’s comparing number of self-reported IVF cycles to clinical records were moderate; for all combined cycles (fresh and frozen) K_w_ was 0.50 (95% CI 0.45, 0.55), for fresh cycles only K_w_ was 0.50 (95% CI 0.45, 0.55), and for frozen cycles only K_w_ was 0.40 (95% CI 0.32, 0.49).

**Table 2:** Fertility treatment usage before IVF Study reported at ART-FS compared to self-report at IVF Study

					Cohen’s kappa Κ (95% CI)	Sensitivity (95% CI)	Specificity (95% CI)
**Compared to self-report at IVF study**^**a**^							
Use of IVF	ART-FS recall				0.28 (0.22, 0.33)	0.85 (0.79, 0.92)	0.63 (0.59, 0.67)
	IVF Study recall		+	–			
		+	94	16			
		–	222	382			
Use of Clomiphene or Gonadotropin	ART-FS recall				0.36 (0.28, 0.42)	0.81 (0.78, 0.84)	0.55 (0.48, 0.61)
	IVF Study recall		+	–			
		+	463	109			
		–	107	129			
Use of any fertility treatment	ART-FS recall				0.36 (0.29, 0.43)	0.85 (0.80, 0.86)	0.52 (0.45, 0.58)
	IVF Study recall		+	–			
		+	491	97			
		–	106	114			

*ART-FS* AfteR Treatment Follow-up Study, *IVF* in vitro fertilization, *95% CI* 95% confidence interval

^a^ Self-report at IVF Study enrollment used as gold standard to calculate specificity and sensitivity

**Table 3:** IVF usage during IVF Study reported at ART-FS compared to clinical records^a^

					Cohen’s kappa Κ (95% CI)	Sensitivity (95% CI)	Specificity (95% CI)
**Type of transfer**							
Fresh IVF cycles	ART-FS recall				0.03 (−0.02, 0.08)	0.88 (0.86, 0.90)	0.27 (0.01, 0.54)
	Clinical records		+	–			
		+	700	97			
		–	8	3			
Frozen IVF cycles	ART-FS recall				0.22 (0.15, 0.29)	0.56 (0.49, 0.64)	0.71 (0.68, 0.75)
	Clinical records		+	–			
		+	92	71			
		–	186	459			
					Weighted Cohen’s kappa Κ (95% CI)		
**Number of IVF cycles**							
All (fresh and frozen) (0–6+)					0.50 (0.45, 0.55)		
Fresh (0–6+)					0.50 (0.45, 0.55)		
Frozen (0–6+)					0.40 (0.32, 0.49)		

*ART-FS* AfteR Treatment Follow-up Study, *IVF* in vitro fertilization, *95% CI* 95% confidence interval

^a^ Clinical records used as gold standard to calculate specificity and sensitivity

When evaluating validity of self-reported recall of infertility diagnoses, sensitivity values and K’s were higher among women with a single self-reported infertility diagnosis (*N* = 509) than women with multiple diagnoses (*N* = 808) (Table 4). Among women with a single self-reported infertility diagnosis, recall of all infertility diagnoses had relatively high sensitivity (> 0.61) and specificity (≥ 0.79) (excluding uterine factor infertility which had a small sample size). Male factor infertility (K = 0.82, 95% CI 0.76, 0.87), endometriosis (K = 0.76, 95% CI 0.65, 0.86) and tubal factor infertility (K = 0.73, 95% CI 0.64, 0.82) had the highest agreement between the two self-reported questionnaires.

**Table 4:** Self-reported primary infertility diagnosis at ART-FS compared to self-report from IVF Study^a^

	Women who self-reported a single diagnosis at IVF study enrollment (*n* = 509)							Women who self-reported any number of diagnoses at IVF study enrollment (n = 808)						
Infertility diagnosis					Cohen’s kappa Κ (95% CI)	Sensitivity (95% CI)	Specificity (95% CI)					Cohen’s kappa Κ (95% CI)	Sensitivity (95% CI)	Specificity (95% CI)
Any female factor infertility^b^	ART-FS recall				0.61 (0.54, 0.68)	0.87 (0.81, 0.92)	0.79 (0.74, 0.83)	ART-FS recall				0.51 (0.45, 0.57)	0.78 (0.73, 0.82)	0.74 (0.70, 0.78)
	IVF Study recall		+	–				IVF Study recall		+	–			
		+	148	23					+	273	79			
		–	70	257					–	114	325			
PCOS	ART-FS recall				0.66 (0.51, 0.81)	0.72 (0.54, 0.90)	0.98 (0.97, 0.99)	ART-FS recall				0.53 (0.42, 0.64)	0.48 (0.37, 0.60)	0.98 (0.97, 0.99)
	IVF Study recall		+	–				IVF Study recall		+	–			
		+	18	7					+	34	36			
		–	10	463					–	16	705			
Endometriosis	ART-FS recall				0.76 (0.65, 0.86)	0.76 (0.62, 0.89)	0.98 (0.97, 0.99)	ART-FS recall				0.60 (0.51, 0.68)	0.53 (0.44, 0.61)	0.98 (0.97, 0.99)
	IVF Study recall		+	–				IVF Study recall		+	–			
		+	31	10					+	68	61			
		–	8	449					–	13	647			
Uterine factor infertility	ART-FS recall				0.07 (−0.09, 0.22)	0.17 (0.0, 0.46)	0.97 (0.95, 0.98)	ART-FS recall				0.14 (0.01, 0.26)	0.16 (0.04, 0.28)	0.96 (0.95, 0.98)
	IVF Study recall		+	–				IVF Study recall		+	–			
		+	1	5					+	6	31			
		–	17	475					–	25	702			
Tubal factor infertility	ART-FS recall				0.73 (0.64, 0.82)	0.66 (0.56, 0.77)	0.99 (0.98, 1.00)	ART-FS recall				0.59 (0.51, 0.67)	0.50 (0.42, 0.58)	0.99 (0.98, 1.00)
	IVF Study recall		+	–				IVF Study recall		+	–			
		+	53	27					+	76	77			
		–	5	413					–	6	632			
Diminished ovarian reserve/ Increased maternal age	ART-FS recall				0.25 (0.14, 0.37)	0.74 (0.54, 0.93)	0.87 (0.84, 0.90)	ART-FS recall				0.31 (0.21, 0.40)	0.65 (0.51, 0.79)	0.87 (0.85, 0.90)
	IVF Study recall		+	–				IVF Study recall		+	–			
		+	14	5					+	30	16			
		–	61	418					–	86	585			
Male factor infertility	ART-FS recall				0.82 (0.76, 0.87)	0.82 (0.76, 0.87)	0.97 (0.96, 0.99)	ART-FS recall				0.65 (0.60, 0.71)	0.64 (0.59, 0.69)	0.98 (0.96, 0.99)
	IVF Study recall		+	–				IVF Study recall		+	–			
		+	149	33					+	196	110			
		–	8	308					–	12	471			
Other/ Unknown	ART-FS recall				0.55 (0.47, 0.63)	0.62 (0.54, 0.70)	0.91 (0.88, 0.94)	ART-FS recall				0.34 (0.27, 0.41)	0.48 (0.41, 0.54)	0.85 (0.82, 0.88)
	IVF Study recall		+	–				IVF Study recall		+	–			
		+	90	55					+	115	127			
		–	33	320					–	84	470			

*ART-FS* AfteR Treatment Follow-up Study, *PCOS* polycystic ovarian syndrome

^a^ Self-report at IVF Study enrollment used as gold standard to calculate specificity and sensitivity

^b^ Any female factor infertility includes at least one of: PCOS, endometriosis, uterine factor infertility, tubal factor infertility, or diminished reserve/advanced age

In general, the agreement between self-reported primary infertility diagnosis from the ART-FS and clinical records (Table 5) was not as strong as the agreement with self-report at IVF Study enrollment (Table 4). Restriction to the primary clinical diagnosis had higher sensitivity and K’s in comparison to values calculated when considering any diagnosis from the medical records (Table 5). However, the improvements were not large, and values of several diagnoses were unchanged (e.g. PCOS, uterine factor infertility, and diminished ovarian reserve/increased maternal age).

**Table 5:** Self-reported primary infertility diagnosis at ART-FS compared to clinical record^a^

	Primary diagnosis from clinical records only							Any diagnosis from clinical record (primary, secondary, other)						
Infertility diagnosis					Cohen’s kappa Κ (95% CI)	Sensitivity (95% CI)	Specificity (95% CI)					Cohen’s kappa Κ (95% CI)	Sensitivity (95% CI)	Specificity (95% CI)
Any female factor infertility^b^	ART-FS recall				0.41 (0.35, 0.48)	0.72 (0.67, 0.76)	0.70 (0.66, 0.74)	ART-FS recall				0.37 (0.30, 0.43)	0.66 (0.61, 0.70)	0.71 (0.67, 0.76)
	Clinical records		+	–				Clinical records		+	–			
		+	256	102					+	281	146			
		–	130	303					–	105	259			
PCOS	ART-FS recall				0.31 (0.18, 0.44)	0.39 (0.24, 0.54)	0.95 (0.94, 0.97)	ART-FS recall				0.35 (0.23, 0.48)	0.38 (0.25, 0.51)	0.96 (0.94, 0.97)
	Clinical records		+	–				Clinical records		+	–			
		+	16	25					+	21	30			
		–	35	715					–	34	706			
Endometriosis	ART-FS recall				0.48 (0.38, 0.57)	0.46 (0.37, 0.56)	0.96 (0.94, 0.97)	ART-FS recall				0.42 (0.34, 0.51)	0.38 (0.30, 0.46)	0.96 (0.95, 0.98)
	Clinical records		+	–				Clinical records		+	–			
		+	51	59					+	58	94			
		–	29	652					–	22	617			
Uterine factor infertility	ART-FS recall				0.01 (−0.07, 0.09)	0.06 (< 0.01, 0.16)	0.96 (0.95, 0.97)	ART-FS recall				0.02 (−0.06, 0.10)	0.06 (< 0.01, 0.14)	0.96 (0.94, 0.97)
	Clinical records		+	–				Clinical records		+	–			
		+	1	17					+	2	32			
		–	31	742					–	30	727			
Tubal factor infertility	ART-FS recall				0.62 (0.54, 0.70)	0.54 (0.46 0.63)	0.98 (0.98, 0.99)	ART-FS recall				0.39 (0.32, 0.46)	0.33 (0.27, 0.39)	0.99 (0.98, 1.00)
	Clinical records		+	–				Clinical records		+	–			
		+	72	61					+	76	157			
		–	10	648					–	6	552			
Diminished ovarian reserve/ Increased maternal age	ART-FS recall				0.07 (<−0.01, 0.14)	0.28 (0.17, 0.40)	0.83 (0.80, 0.86)	ART-FS recall				0.06 (−0.01, 0.14)	0.25 (0.16, 0.35)	0.83 (0.80, 0.86)
	Clinical records		+	–				Clinical records		+	–			
		+	16	40					+	21	62			
		–	125	610					–	120	588			
Male factor infertility		ART-FS recall			0.66 (0.61, 0.72)	0.67 (0.61, 0.72)	0.96 (0.94, 0.97)		ART-FS recall			0.61 (0.55, 0.66)	0.60 (0.55, 0.65)	0.97 (0.96, 0.99)
	Clinical records		+	–				Clinical records		+	–			
		+	184	91					+	195	130			
		–	23	493					–	12	454			
Other/ Unknown	ART-FS recall				0.30 (0.22, 0.38)	0.51 (0.43, 0.59)	0.82 (0.78, 0.84)	ART-FS recall				0.29 (0.21, 0.36)	0.49 (0.41, 0.56)	0.82 (0.78, 0.85)
	Clinical records		+	–				Clinical records		+	–			
		+	81	77					+	84	88			
		–	117	516					–	114	505			

*ART-FS* AfteR Treatment Follow-up Study, *PCOS* polycystic ovarian syndrome

^a^ Self-report at IVF Study enrollment used as gold standard to calculate specificity and sensitivity

^b^ Any female factor infertility includes at least one of: PCOS, endometriosis, uterine factor infertility, tubal factor infertility, or diminished reserve/advanced age

The recall of details of IVF cycles during the IVF Study (type of transfer, number of cycles) among those who received additional IVF treatments after the IVF study compared to recall of those who did not receive additional IVF treatments were generally the same (Supplemental Table 1). When we repeated our main analyses of infertility diagnoses (Tables 4 and 5) after excluding women who received additional IVF treatments after the IVF Study, the results were generally unchanged (Supplemental Tables 2–3). Similarly, when we instead excluded women who had more than two IVF cycles during the IVF Study, the results were generally unchanged compared to the results from our main analyses (Supplemental Tables 4–5).

## Discussion

### Principal findings

We observed that approximately 20 years after fertility treatment, women’s recall of a specific period of their treatment history varied greatly by the level of treatment detail, while recall of their primary infertility diagnosis varied by diagnosis. Recall of self-reported use of fertility treatment had consistently moderate sensitivity but low specificity across different infertility treatment modalities. Recalled details of IVF cycles (number of cycles, fresh or frozen embryo transfers) had low to moderate validity and reliability compared with medical records. We found that accuracy of primary infertility diagnosis recall was higher for self-report compared to medical records. Validity and reliability for primary infertility diagnosis also varied greatly depending on the diagnosis.

### Interpretation

Prior research focused on the validity and reliability of recalled fertility treatment and infertility diagnoses has been sparse with limited duration of follow up. In a previous study by Thomas et al. [14], 63 women receiving services from a specialized fertility treatment center in 2004 reported that elements of women’s fertility treatment history could be accurately captured (more than 90% sensitivity for all elements) by a self-reported questionnaire, 5–6 years after treatment initiation [14]. Research from the Nurses’ Health Study II, also supports this finding, and found > 80% concordance of self-reported gonadotropin use when comparing prospective reports to lifetime history with a maximum of 16 years of follow-up [30]. In our study, the correlation between self-report of ever use of IVF and medical records was high (K = 0.74, 95% CI 0.57, 0.90; sensitivity = 0.96, 95% CI 0.88, 1.00; specificity = 0.82, 95% CI 0.69, 0.94). In comparison, we observed low to moderate validity and reliability between self-reported treatment history at follow-up and self-reported treatment history at original study initiation. The lower values that we detected could be due to several factors. In our study, participants were asked to recall details an average of 20 years after treatment initiation (approximately 15 years longer than other studies). It has been shown for other health conditions that self-report is subject to recall bias, particularly with increasing duration between the event and the survey [31]. Our results also examined precise treatment details (treatment during clearly defined time periods, number of cycles, fresh versus frozen embryo cycles). To our knowledge, this is the first study to examine these details of fertility treatment history. However, the complexity of these details may represent a barrier to recall given the assumed health literacy necessary to recall accurately. This level of information may not be appropriate to utilize in studies involving participants from the general public. The questionnaire developed by Thomas et al. prefaced sections on various fertility treatments with introductory sentences defining the treatment modality in clear terms (e.g. “…By ART treatment, we mean any treatment that involves removing the egg from the woman’s body and then replacing the egg or embryo back into the body”) and to capture pregnancies and attempts to conceive, provided an extensive definition for an “attempt” and multiple examples of responses using their definitions for different scenarios. Therefore, future investigators could consider asking about a woman’s fertility history more broadly and provide definitions or examples for critical items of interest to capture more accurate information, especially over an extended period of recall.

In our study, we observed that accuracy of infertility diagnosis at follow-up was higher when compared to self-report at treatment initiation than when compared to medical records. To our knowledge, this is the first study to report comparisons between to self-report at prior study enrollment and medical records. The higher validity and reliability across self-report could suggest that there are differences in the way that women interpret or attribute cause to their infertility compared to clinicians. This may have implications for clinical practice and clinicians may consider ensuring diagnoses and results are more clearly communicated to patients.

Our analyses of primary infertility diagnosis also revealed great variability in validity and reliability depending on the specific diagnosis. It is possible that participants could have reported a secondary instead of their primary diagnosis during the ART-FS due to recall issues, however, in analyses where we considered women with one or more infertility diagnoses during the IVF Study (Tables 4 and 5), recall was not improved. It is also plausible that women who have unsuccessful fertility treatment attempts may receive additional infertility diagnoses as their treatment progresses. However, in sensitivity analyses where we (i) excluded women who reported receiving additional IVF treatments after the IVF Study or (ii) excluded women who received more than two IF cycles during the IVF Study, recall was not improved compared to our main results (Tables 4 and 5). Highest values comparing self-report to clinical records in our study were seen for primary diagnoses of male factor infertility (K = 0.66, 95% CI 0.61, 0.72; sensitivity = 0.67, 95% CI 0.61, 0.72; specificity = 0.96, 95% CI 0.94, 0.97) and tubal factor infertility (K = 0.62, 95% CI 0.54, 0.70; sensitivity = 0.54, 95% CI 0.46, 0.63; specificity = 0.98, 95% CI 0.98, 0.99). A study by de Boer et al. [20], comparing self-reported diagnoses to medical records in the Netherlands, also reported that the highest validity and reliability values were seen for a diagnosis of either male factor (K = 0.71; sensitivity = 0.78; specificity = 0.91) or tubal factor infertility (K = 0.79; sensitivity = 0.84; specificity = 0.94). Male factor and tubal factor infertility may have a more clearly defined etiology and therefore have higher accuracy of recall, compared to less prevalent and complex factors such as hormone-related infertility. De Boer et al. observed that fewer than 18% of participants had 8 or more years of follow-up [20] while in our study the average time between recall and treatment initiation was almost 20 years. The greater period of follow-up combined with the differences in measurement of infertility in our study’s medical records compared to the ART-FS questionnaire may have contributed to the overall lower values of validity and reliability compared to de Boer et al. [20]. This suggests that investigators who are planning a study involving infertility diagnosis recalled over an extended time should consider providing more details about or specific examples of the infertility categories they are interested in capturing.

### Strengths and limitations

The ART-FS was formed from a previous cohort of women who sought IVF services approximately 20 years ago, which to our knowledge, is the longest period of follow-up with detailed self-report and medical record data available in the current literature [14, 20]. Our study accessed extensive clinical records from a prior IVF study, allowing us to consider the accuracy of recalled details of fertility treatment (fertility treatments during a specific timeframe, number of cycles, fresh versus frozen embryo transfers) that had not been considered by previous studies. Additionally, we were able to evaluate the accuracy of self-reported infertility and treatment at follow-up compared to self-report at treatment initiation, which to our knowledge has not yet been reported.

Despite these strengths, there are several important limitations to our study that should be considered. There is potential misclassification of infertility diagnosis due to the different terminology used across the medical records and two separate questionnaires. As noted previously, this may affect less prevalent diagnoses and/or diagnoses with more complex etiology or diagnostic criteria (e.g. uterine factor infertility, diminished ovarian reserve/increased maternal age) more so than other more specific diagnoses (e.g. tubal factor or male factor infertility). During the ART-FS, we only asked participants to report their primary infertility diagnosis, while at treatment initiation and in medical records, multiple diagnoses could be recorded. As a result, while we were able to successfully consider women with a singular diagnosis, we were not able to effectively evaluate women with multiple diagnoses. Indeed, when we restricted our sample sizes to women who either only self-reported one diagnosis (at treatment initiation) or only had a primary infertility diagnosis (in medical record), validity and reliability values increased. In addition, changes in infertility diagnoses or clinical diagnosis procedures compared to when our cohort began fertility treatments (1994–2003) may reduce generalizability compared to current treatment standards.

It should also be noted that the women who did participate in our analysis differed with regards to certain characteristics from the women who we were either not able to recontact or who chose to not participate in the ART-FS. Women in the ART-FS were more likely to be non-Hispanic white and to have at least a college degree. These women were also more likely to have had a successful IVF cycle during the IVF study (55%) compared to women who chose not to participate (31%) and women who we were not able to recontact (46%). These differences may affect our ability to generalize our results to other groups of women utilizing infertility treatments. It is possible that women who were less fixated on the outcome of their IVF cycles during the IVF Study were less likely to accurately recall the details of their treatment. For example, women who experienced a successful IVF cycle could have been more satisfied with their treatment and less likely to recall the details of their treatment in the same way as women who did not have a successful IVF cycle and therefore, may have been less satisfied with their treatment. Few studies that have investigated the potential association between patient perception/experience during clinical interactions with their recall ability have produced mixed results [32, 33] and recent evidence is lacking.

## Conclusions

In order to use women’s self-reported fertility data for research purposes we must have confidence that this information is recalled and reported accurately. Our study examining women’s recall of their infertility and treatment history almost 20 years after their fertility treatment initiation shows that women previously treated for infertility are moderately accurate in their recall very specific treatment details. Reliability of self-reported infertility diagnosis varied by diagnosis and method of measurement. Researchers should consider these issues when designing studies and utilizing self-reported history of infertility to improve the accuracy of measurement collection.

## Supplementary Information

**Additional file 1: Supplemental Tables 1–5.** Results of sensitivity analyses.

## Data Availability

The datasets generated and/or analysed during the current study are not publicly available due to restrictions on sharing data, based the consent forms and IRB application for this study, but are available from the corresponding author on reasonable request.

## Abbreviations

95% CI: 95% confidence interval
ART: Assisted reproductive technology
ART-FS: AfteR Treatment Follow-up Study
GIFT: Gamete intrafallopian transfer
IVF: In vitro fertilization
K: Cohen’s kappa
K_w_: Weighted Cohen’s Kappa
PCOS: Polycystic ovarian syndrome
REDCap: Research Electronic Data Capture
SD: Standard deviation
