# Supplementary material for: Self-reported infertility diagnoses and treatment history approximately 20 years after fertility treatment initiation
Source: Fertil Res Pract. 2021 Mar 12;7:7. doi: 10.1186/s40738-021-00099-2 (PMC7953690; doi:10.1186/s40738-021-00099-2)
Supplement: Supplementary file 1 — Additional file 1: Supplemental Tables 1–5. Results of sensitivity analyses. [file 40738_2021_99_MOESM1_ESM.docx]

| **Supplemental Table 1. IVF usage during IVF Study reported at ART-FS by IVF usage after IVF Study^a^** | | | | | | | | | | | | | | |
| --- | --- | --- | --- | --- | --- | --- | --- | --- | --- | --- | --- | --- | --- | --- |
|  | Women who did receive IVF after participation in IVF Study | | | | | | | Women who did not receive IVF after participation in IVF Study | | | | | | |
|  |  | | | | Cohen’s kappa  Κ (95% CI) | Sensitivity  (95% CI) | Specificity  (95% CI) |  | | | | Cohen’s kappa  Κ (95% CI) | Sensitivity  (95% CI) | Specificity  (95% CI) |
| **Type of transfer** | | | | |  |  |  |  | | | |  |  |  |
| Fresh IVF cycles | ART-FS recall | | | | 0.06  (-0.09, 0.20) | 0.89  (0.85, 0.94) | 0.33  (0, 0.87) | ART-FS recall | | | | 0.03  (-0.03, 0.09) | 0.86  (0.83, 0.89) | 0.40  (0, 0.83) |
|  | Clinical records |  | + | - |  |  |  | Clinical records |  | + | - |  |  |  |
|  |  | + | 1 | 2 |  |  |  |  | + | 2 | 3 |  |  |  |
|  |  | - | 20 | 170 |  |  |  |  | - | 73 | 438 |  |  |  |
| Frozen IVF cycles | ART-FS recall | | | | 0.27  (0.09, 0.45) | 0.67  (0.49, 0.84) | 0.65  (0.55, 0.76) | ART-FS recall | | | | 0.24  (0.53, 0.33) | 0.43  (0.33, 0.52) | 0.83  (0.80, 0.86) |
|  | Clinical records |  | + | - |  |  |  | Clinical records |  | + | - |  |  |  |
|  |  | + | 49 | 26 |  |  |  |  | + | 401 | 82 |  |  |  |
|  |  | - | 9 | 18 |  |  |  |  | - | 60 | 45 |  |  |  |
|  |  | | | | Weighted Cohen’s kappa  Κ_w_ (95% CI) |  |  |  | | | | Weighted Cohen’s kappa  Κ_w_ (95% CI) |  |  |
| **Number of IVF cycles** | | | | |  |  |  |  | | | |  |  |  |
| All (fresh and frozen) (0-6+) | | | | | 0.51  (0.43, 0.59) |  |  |  | | | | 0.52  (0.46, 0.58) |  |  |
| Fresh (0-6+) | | | | | 0.48  (0.39, 0.56) |  |  |  | | | | 0.52  (0.46, 0.58) |  |  |
| Frozen (0-6+) | | | | | 0.49  (0.30, 0.68) |  |  |  | | | | 0.36  (0.26, 0.46) |  |  |
| ART-FS= AfteR Treatment Follow-up Study; IVF= in vitro fertilization; PPV= positive predictive value; NPV= negative predictive value; CI= confidence interval  ^a^ Clinical record used as gold standard to calculate specificity and sensitivity | | | | | | | | | | | | | | |

| **Supplemental Table 2. Self-reported primary infertility diagnosis at ART-FS compared to self-reported diagnosis at IVF Study enrollment, among women with no additional IVF after IVF Study ^a^** | | | | | | | | | | | | | | |
| --- | --- | --- | --- | --- | --- | --- | --- | --- | --- | --- | --- | --- | --- | --- |
|  | Women who self-reported a single diagnosis at IVF Study and did not receive additional IVF after IVF Study  (N=301) | | | | | | | Women who self-reported any number of diagnoses at IVF Study and did not receive additional IVF after IVF Study  (N=484) | | | | | | |
| **Infertility diagnosis** |  | | | | Cohen’s kappa  Κ (95% CI) | Sensitivity  (95% CI) | Specificity  (95% CI) |  | | | | Cohen’s kappa  Κ (95% CI) | Sensitivity  (95% CI) | Specificity  (95% CI) |
| Any female factor infertility^b^ | ART-FS recall | | | | 0.63  (0.55, 0.72) | 0.92  (0.86, 0.97) | 0.76  (0.70, 0.82) | ART-FS recall | | | | 0.51  (0.44, 0.59) | 0.79  (0.74, 0.85) | 0.72  (0.67, 0.78) |
|  | IVF Study recall |  | + | - |  |  |  | IVF Study recall |  | + | - |  |  |  |
|  |  | + | 99 | 9 |  |  |  |  | + | 176 | 46 |  |  |  |
|  |  | - | 45 | 144 |  |  |  |  | - | 71 | 185 |  |  |  |
| PCOS | ART-FS recall | | | | 0.57  (0.35, 0.80) | 0.57  (0.31, 0.83) | 0.98  (0.97,1.00) | ART-FS recall | | | | 0.53  (0.38, 0.67) | 0.46  (0.31, 0.62) | 0.98  (0.97, 0.99) |
|  | IVF Study recall |  | + | - |  |  |  | IVF Study recall |  | + | - |  |  |  |
|  |  | + | 8 | 6 |  |  |  |  | + | 19 | 22 |  |  |  |
|  |  | - | 5 | 278 |  |  |  |  | - | 8 | 429 |  |  |  |
| Endometriosis | ART-FS recall | | | | 0.77  (0.64, 0.90) | 0.88  (0.74, 1.00) | 0.97  (0.95, 0.99) | ART-FS recall | | | | 0.60  (0.49, 0.70) | 0.56  (0.45, 0.67) | 0.97  (0.95, 0.99) |
|  | IVF Study recall |  | + | - |  |  |  | IVF Study recall |  | + | - |  |  |  |
|  |  | + | 21 | 3 |  |  |  |  | + | 43 | 34 |  |  |  |
|  |  | - | 8 | 265 |  |  |  |  | - | 12 | 389 |  |  |  |
| Uterine factor infertility | ART-FS recall | | | | 0.11  (-0.11, 0.33) | 0.25  (0, 0.67) | 0.96  (0.94, 0.98) | ART-FS recall | | | | 0.19  (0.02, 0.36) | 0.20  (0.04, 0.36) | 0.97  (0.95, 0.98) |
|  | IVF Study recall |  | + | - |  |  |  | IVF Study recall |  | + | - |  |  |  |
|  |  | + | 1 | 3 |  |  |  |  | + | 5 | 20 |  |  |  |
|  |  | - | 11 | 282 |  |  |  |  | - | 14 | 425 |  |  |  |
| Tubal factor infertility | ART-FS recall | | | | 0.76  (0.66, 0.87) | 0.69  (0.56, 0.81) | 0.99  (0.98, 1.00) | ART-FS recall | | | | 0.61  (0.52, 0.71) | 0.52  (0.41, 0.62) | 0.99  (0.98, 1.00) |
|  | IVF Study recall |  | + | - |  |  |  | IVF Study recall |  | + | - |  |  |  |
|  |  | + | 35 | 16 |  |  |  |  | + | 48 | 45 |  |  |  |
|  |  | - | 2 | 244 |  |  |  |  | - | 3 | 382 |  |  |  |
| Diminished ovarian reserve/ Increased maternal age | ART-FS recall | | | | 0.30  (0.16, 0.44) | 0.80  (0.60, 1.00) | 0.85  (0.81, 0.90) | ART-FS recall | | | | 0.37  (0.25, 0.49) | 0.71  (0.56, 0.86) | 0.86  (0.83, 0.90) |
|  | IVF Study recall |  | + | - |  |  |  | IVF Study recall |  | + | - |  |  |  |
|  |  | + | 12 | 3 |  |  |  |  | + | 25 | 10 |  |  |  |
|  |  | - | 41 | 241 |  |  |  |  | - | 54 | 343 |  |  |  |
| Male factor infertility | ART-FS recall | | | | 0.84  (0.78, 0.91) | 0.84  (0.77, 0.92) | 0.98  (0.95, 1.00) | ART-FS recall | | | | 0.68  (0.61, 0.75) | 0.66  (0.59, 0.74) | 0.97  (0.96, 0.99) |
|  | IVF Study recall |  | + | - |  |  |  | IVF Study recall |  | + | - |  |  |  |
|  |  | + | 81 | 15 |  |  |  |  | + | 111 | 56 |  |  |  |
|  |  | - | 5 | 196 |  |  |  |  | - | 8 | 303 |  |  |  |
| Other/ Unknown | ART-FS recall | | | | 0.56  (0.46, 0.66) | 0.58  (0.48, 0.68) | 0.94  (0.90, 0.97) | ART-FS recall | | | | 0.31  (0.21, 0.40) | 0.43  (0.35, 0.51) | 0.86  (0.82, 0.89) |
|  | IVF Study recall |  | + | - |  |  |  | IVF Study recall |  | + | - |  |  |  |
|  |  | + | 54 | 39 |  |  |  |  | + | 66 | 88 |  |  |  |
|  |  | - | 13 | 191 |  |  |  |  | - | 47 | 278 |  |  |  |
| ART-FS= AfteR Treatment Follow-up Study; PCOS= polycystic ovarian syndrome  ^a^ Self-report at IVF Study enrollment used as gold standard to calculate specificity and sensitivity  ^b^ Any female factor infertility includes at least one of: PCOS, endometriosis, uterine factor infertility, tubal factor infertility, or diminished reserve/advanced age. | | | | | | | | | | | | | | |

| **Supplemental Table 3. Self-reported primary infertility diagnosis at ART-FS compared to clinical record, among women with no additional IVF after IVF Study, N=484^a^** | | | | | | | | | | | | | | |
| --- | --- | --- | --- | --- | --- | --- | --- | --- | --- | --- | --- | --- | --- | --- |
|  | Primary diagnosis from clinical records of women who did not receive additional IVF after IVF Study | | | | | | | Any diagnosis from clinical record (primary, secondary, other) of women who did not receive additional IVF after IVF Study | | | | | | |
| **Infertility diagnosis** |  | | | | Cohen’s kappa  Κ (95% CI) | Sensitivity  (95% CI) | Specificity  (95% CI) |  | | | | Cohen’s kappa  Κ (95% CI) | Sensitivity  (95% CI) | Specificity  (95% CI) |
| Any female factor infertility^b^ | ART-FS recall | | | | 0.43  (0.34, 0.51) | 0.74  (0.68, 0.79) | 0.69  (0.63, 0.75) | ART-FS recall | | | | 0.36  (0.28, 0.44) | 0.68  (0.62, 0.73) | 0.69  (0.62, 0.75) |
|  | Clinical records |  | + | - |  |  |  | Clinical records |  | + | - |  |  |  |
|  |  | + | 169 | 60 |  |  |  |  | + | 180 | 86 |  |  |  |
|  |  | - | 77 | 170 |  |  |  |  | - | 66 | 144 |  |  |  |
| PCOS | ART-FS recall | | | | 0.27  (0.10, 0.44) | 0.32  (0.14, 0.50) | 0.96  (0.94, 0.98) | ART-FS recall | | | | 0.30  (0.13, 0.46) | 0.31  (0.15, 0.47) | 0.96  (0.94, 0.98) |
|  | Clinical records |  | + | - |  |  |  | Clinical records |  | + | - |  |  |  |
|  |  | + | 8 | 17 |  |  |  |  | + | 10 | 22 |  |  |  |
|  |  | - | 19 | 432 |  |  |  |  | - | 17 | 427 |  |  |  |
| Endometriosis | ART-FS recall | | | | 0.48  (0.37, 0.60) | 0.49  (0.37, 0.60) | 0.95  (0.93, 0.97) | ART-FS recall | | | | 0.40  (0.30, 0.51) | 0.38  (0.28, 0.48) | 0.95  (0.93, 0.98) |
|  | Clinical records |  | + | - |  |  |  | Clinical records |  | + | - |  |  |  |
|  |  | + | 35 | 37 |  |  |  |  | + | 38 | 62 |  |  |  |
|  |  | - | 20 | 384 |  |  |  |  | - | 17 | 359 |  |  |  |
| Uterine factor infertility | ART-FS recall | | | | 0.06  (-0.09, 0.20) | 0.14  (0, 0.40) | 0.96  (0.94, 0.98) | ART-FS recall | | | | 0.02  (-0.09, 0.13) | 0.06  (0, 0.17) | 0.96  (0.94, 0.98) |
|  | Clinical records |  | + | - |  |  |  | Clinical records |  | + | - |  |  |  |
|  |  | + | 1 | 6 |  |  |  |  | + | 1 | 16 |  |  |  |
|  |  | - | 18 | 451 |  |  |  |  | - | 18 | 441 |  |  |  |
| Tubal factor infertility | ART-FS recall | | | | 0.63  (0.53, 0.73) | 0.54  (0.44, 0.65) | 0.99  (0.98, 1.00) | ART-FS recall | | | | 0.37  (0.29, 0.46) | 0.32  (0.24, 0.39) | 0.99  (0.98, 1.00) |
|  | Clinical records |  | + | - |  |  |  | Clinical records |  | + | - |  |  |  |
|  |  | + | 47 | 40 |  |  |  |  | + | 49 | 106 |  |  |  |
|  |  | - | 4 | 385 |  |  |  |  | - | 2 | 319 |  |  |  |
| Diminished ovarian reserve/ Increased maternal age | ART-FS recall | | | | 0.11  (0.01, 0.21) | 0.37  (0.22, 0.52) | 0.82  (0.78, 0.85) | ART-FS recall | | | | 0.08  (-0.02, 0.18) | 0.29  (0.17, 0.41) | 0.81  (0.78, 0.85) |
|  | Clinical records |  | + | - |  |  |  | Clinical records |  | + | - |  |  |  |
|  |  | + | 14 | 24 |  |  |  |  | + | 16 | 39 |  |  |  |
|  |  | - | 80 | 358 |  |  |  |  | - | 78 | 343 |  |  |  |
| Male factor infertility |  | ART-FS recall | | | 0.66  (0.59, 0.74) | 0.68  (0.60, 0.75) | 0.95  (0.92, 0.97) |  | ART-FS recall | | | 0.62  (0.55, 0.69) | 0.61  (0.54, 0.68) | 0.97  (0.95, 0.99) |
|  | Clinical records |  | + | - |  |  |  | Clinical records |  | + | - |  |  |  |
|  |  | + | 101 | 48 |  |  |  |  | + | 109 | 70 |  |  |  |
|  |  | - | 17 | 310 |  |  |  |  | - | 9 | 288 |  |  |  |
| Other/ Unknown | ART-FS recall | | | | 0.30  (0.20, 0.40) | 0.49  (0.39, 0.59) | 0.83  (0.79, 0.87) | ART-FS recall | | | | 0.29  (0.19, 0.39) | 0.46  (0.37, 0.56) | 0.83  (0.79, 0.87) |
|  | Clinical records |  | + | - |  |  |  | Clinical records |  | + | - |  |  |  |
|  |  | + | 48 | 50 |  |  |  |  | + | 49 | 57 |  |  |  |
|  |  | - | 64 | 314 |  |  |  |  | - | 63 | 307 |  |  |  |
| ART-FS= AfteR Treatment Follow-up Study; PCOS= polycystic ovarian syndrome  ^a^ Self-report at IVF Study enrollment used as gold standard to calculate specificity and sensitivity  ^b^ Any female factor infertility includes at least one of: PCOS, endometriosis, uterine factor infertility, tubal factor infertility, or diminished reserve/advanced age. | | | | | | | | | | | | | | |

| **Supplemental Table 4. Self-reported primary infertility diagnosis at ART-FS compared to self-reported diagnosis at IVF Study enrollment, among women who received ≤ 2 IVF cycles during IVF Study^a^** | | | | | | | | | | | | | | |
| --- | --- | --- | --- | --- | --- | --- | --- | --- | --- | --- | --- | --- | --- | --- |
|  | Women who self-reported a single diagnosis at IVF Study and received ≤ 2 IVF cycles during IVF Study  (N=392) | | | | | | | Women who self-reported any number of diagnoses at IVF Study and received ≤ 2 IVF cycles during IVF Study  (N=615) | | | | | | |
| **Infertility diagnosis** |  | | | | Cohen’s kappa  Κ (95% CI) | Sensitivity  (95% CI) | Specificity  (95% CI) |  | | | | Cohen’s kappa  Κ (95% CI) | Sensitivity  (95% CI) | Specificity  (95% CI) |
| Any female factor infertility^b^ | ART-FS recall | | | | 0.60  (0.52, 0.68) | 0.86  (0.80, 0.92) | 0.79  (0.74, 0.84) | ART-FS recall | | | | 0.48  (0.41, 0.55) | 0.75  (0.70, 0.81) | 0.74  (0.69, 0.78) |
|  | IVF Study recall |  | + | - |  |  |  | IVF Study recall |  | + | - |  |  |  |
|  |  | + | 108 | 18 |  |  |  |  | + | 195 | 64 |  |  |  |
|  |  | - | 55 | 202 |  |  |  |  | - | 90 | 253 |  |  |  |
| PCOS | ART-FS recall | | | | 0.66  (0.50, 0.83) | 0.68  (0.49, 0.88) | 0.98  (0.97, 0.99) | ART-FS recall | | | | 0.52  (0.39, 0.64) | 0.47  (0.34, 0.59) | 0.98  (0.97, 0.99) |
|  | IVF Study recall |  | + | - |  |  |  | IVF Study recall |  | + | - |  |  |  |
|  |  | + | 15 | 7 |  |  |  |  | + | 27 | 31 |  |  |  |
|  |  | - | 7 | 354 |  |  |  |  | - | 12 | 532 |  |  |  |
| Endometriosis | ART-FS recall | | | | 0.72  (0.59, 0.86) | 0.73  (0.58, 0.89) | 0.98  (0.97, 0.99) | ART-FS recall | | | | 0.58  (0.49, 0.68) | 0.51  (0.41, 0.61) | 0.98  (0.97, 0.99) |
|  | IVF Study recall |  | + | - |  |  |  | IVF Study recall |  | + | - |  |  |  |
|  |  | + | 22 | 8 |  |  |  |  | + | 49 | 47 |  |  |  |
|  |  | - | 7 | 346 |  |  |  |  | - | 10 | 496 |  |  |  |
| Uterine factor infertility | ART-FS recall | | | | 0.09  (-0.11, 0.29) | 0.20  (0, 0.55) | 0.97  (0.95, 0.99) | ART-FS recall | | | | 0.12  (-0.02, 0.26) | 0.14  (0.01, 0.27) | 0.97  (0.95, 0.98) |
|  | IVF Study recall |  | + | - |  |  |  | IVF Study recall |  | + | - |  |  |  |
|  |  | + | 1 | 4 |  |  |  |  | + | 4 | 24 |  |  |  |
|  |  | - | 12 | 366 |  |  |  |  | - | 18 | 535 |  |  |  |
| Tubal factor infertility | ART-FS recall | | | | 0.80  (0.71, 0.89) | 0.75  (0.64, 0.87) | 0.99  (0.98, 1.00) | ART-FS recall | | | | 0.62  (0.53, 0.71) | 0.52  (0.43, 0.62) | 0.99  (0.98, 1.00) |
|  | IVF Study recall |  | + | - |  |  |  | IVF Study recall |  | + | - |  |  |  |
|  |  | + | 40 | 13 |  |  |  |  | + | 57 | 52 |  |  |  |
|  |  | - | 4 | 326 |  |  |  |  | - | 4 | 489 |  |  |  |
| Diminished ovarian reserve/ Increased maternal age | ART-FS recall | | | | 0.29  (0.15, 0.43) | 0.75  (0.54, 0.96) | 0.88  (0.85, 0.92) | ART-FS recall | | | | 0.31  (0.19, 0.42) | 0.64  (0.47, 0.80) | 0.88  (0.85, 0.91) |
|  | IVF Study recall |  | + | - |  |  |  | IVF Study recall |  | + | - |  |  |  |
|  |  | + | 12 | 4 |  |  |  |  | + | 21 | 12 |  |  |  |
|  |  | - | 43 | 324 |  |  |  |  | - | 60 | 451 |  |  |  |
| Male factor infertility | ART-FS recall | | | | 0.85  (0.80, 0.91) | 0.87  (0.81, 0.92) | 0.97  (0.95, 0.99) | ART-FS recall | | | | 0.70  (0.64, 0.76) | 0.69  (0.63, 0.75) | 0.97  (0.96, 0.99) |
|  | IVF Study recall |  | + | - |  |  |  | IVF Study recall |  | + | - |  |  |  |
|  |  | + | 116 | 18 |  |  |  |  | + | 152 | 69 |  |  |  |
|  |  | - | 7 | 242 |  |  |  |  | - | 10 | 370 |  |  |  |
| Other/ Unknown | ART-FS recall | | | | 0.56  (0.47, 0.65) | 0.61  (0.52, 0.70) | 0.92  (0.88, 0.95) | ART-FS recall | | | | 0.36  (0.28, 0.44) | 0.49  (0.42, 0.56) | 0.85  (0.82, 0.89) |
|  | IVF Study recall |  | + | - |  |  |  | IVF Study recall |  | + | - |  |  |  |
|  |  | + | 75 | 48 |  |  |  |  | + | 97 | 102 |  |  |  |
|  |  | - | 22 | 238 |  |  |  |  | - | 59 | 347 |  |  |  |
| ART-FS= AfteR Treatment Follow-up Study; PCOS= polycystic ovarian syndrome  ^a^ Self-report at IVF Study enrollment used as gold standard to calculate specificity and sensitivity  ^b^ Any female factor infertility includes at least one of: PCOS, endometriosis, uterine factor infertility, tubal factor infertility, or diminished reserve/advanced age. | | | | | | | | | | | | | | |

| **Supplemental Table 5. Self-reported primary infertility diagnosis at ART-FS compared to clinical record, among women who received ≤ 2 IVF cycles during IVF Study, N=615^a^** | | | | | | | | | | | | | | |
| --- | --- | --- | --- | --- | --- | --- | --- | --- | --- | --- | --- | --- | --- | --- |
|  | Primary diagnosis from clinical records of women who received ≤ 2 IVF cycles during IVF Study | | | | | | | Any diagnosis from clinical record (primary, secondary, other) of women who received ≤ 2 IVF cycles during IVF Study | | | | | | |
| **Infertility diagnosis** |  | | | | Cohen’s kappa  Κ (95% CI) | Sensitivity  (95% CI) | Specificity  (95% CI) |  | | | | Cohen’s kappa  Κ (95% CI) | Sensitivity  (95% CI) | Specificity  (95% CI) |
| Any female factor infertility^b^ | ART-FS recall | | | | 0.42  (0.35, 0.50) | 0.71  (0.66, 0.77) | 0.72  (0.67, 0.76) | ART-FS recall | | | | 0.37  (0.30, 0.45) | 0.65  (0.60, 0.70) | 0.72  (0.67, 0.77) |
|  | Clinical records |  | + | - |  |  |  | Clinical records |  | + | - |  |  |  |
|  |  | + | 189 | 77 |  |  |  |  | + | 204 | 109 |  |  |  |
|  |  | - | 95 | 240 |  |  |  |  | - | 80 | 208 |  |  |  |
| PCOS | ART-FS recall | | | | 0.29  (0.15, 0.44) | 0.38  (0.21, 0.54) | 0.95  (0.93, 0.97) | ART-FS recall | | | | 0.36  (0.22, 0.50) | 0.39  (0.24, 0.53) | 0.96  (0.94, 0.98) |
|  | Clinical records |  | + | - |  |  |  | Clinical records |  | + | - |  |  |  |
|  |  | + | 12 | 20 |  |  |  |  | + | 17 | 27 |  |  |  |
|  |  | - | 28 | 541 |  |  |  |  | - | 23 | 534 |  |  |  |
| Endometriosis | ART-FS recall | | | | 0.49  (0.38, 0.60) | 0.46  (0.36, 0.57) | 0.96  (0.95, 0.98) | ART-FS recall | | | | 0.43  (0.33, 0.52) | 0.38  (0.29, 0.47) | 0.97  (0.95, 0.98) |
|  | Clinical records |  | + | - |  |  |  | Clinical records |  | + | - |  |  |  |
|  |  | + | 39 | 45 |  |  |  |  | + | 43 | 71 |  |  |  |
|  |  | - | 19 | 498 |  |  |  |  | - | 15 | 472 |  |  |  |
| Uterine factor infertility | ART-FS recall | | | | 0.03  (-0.08, 0.13) | 0.08  (0, 0.22) | 0.96  (0.95, 0.98) | ART-FS recall | | | | 0.05  (-0.06, 0.17) | 0.09  (0, 0.21) | 0.96  (0.95, 0.98) |
|  | Clinical records |  | + | - |  |  |  | Clinical records |  | + | - |  |  |  |
|  |  | + | 1 | 12 |  |  |  |  | + | 2 | 20 |  |  |  |
|  |  | - | 22 | 566 |  |  |  |  | - | 21 | 558 |  |  |  |
| Tubal factor infertility | ART-FS recall | | | | 0.66  (0.57, 0.75) | 0.58  (0.48, 0.68) | 0.99  (0.98, 0.99) | ART-FS recall | | | | 0.40  (0.32, 0.48) | 0.34  (0.26, 0.41) | 0.99  (0.98, 1.00) |
|  | Clinical records |  | + | - |  |  |  | Clinical records |  | + | - |  |  |  |
|  |  | + | 54 | 39 |  |  |  |  | + | 56 | 111 |  |  |  |
|  |  | - | 7 | 501 |  |  |  |  | - | 5 | 429 |  |  |  |
| Diminished ovarian reserve/ Increased maternal age | ART-FS recall | | | | 0.08  (<-0.01, 0.17) | 0.30  (0.16, 0.43) | 0.84  (0.81, 0.87) | ART-FS recall | | | | 0.08  (<-0.01, 0.17) | 0.27  (0.15, 0.38) | 0.84  (0.81, 0.87) |
|  | Clinical records |  | + | - |  |  |  | Clinical records |  | + | - |  |  |  |
|  |  | + | 13 | 31 |  |  |  |  | + | 16 | 44 |  |  |  |
|  |  | - | 89 | 468 |  |  |  |  | - | 86 | 455 |  |  |  |
| Male factor infertility |  | ART-FS recall | | | 0.71  (0.65, 0.77) | 0.71  (0.65, 0.78) | 0.96  (0.94, 0.98) |  | ART-FS recall | | | 0.64  (0.57, 0.70) | 0.63  (0.57, 0.69) | 0.97  (0.95, 0.99) |
|  | Clinical records |  | + | - |  |  |  | Clinical records |  | + | - |  |  |  |
|  |  | + | 145 | 58 |  |  |  |  | + | 150 | 88 |  |  |  |
|  |  | - | 16 | 382 |  |  |  |  | - | 11 | 352 |  |  |  |
| Other/ Unknown | ART-FS recall | | | | 0.31  (0.22, 0.39) | 0.52  (0.43, 0.60) | 0.81  (0.78, 0.85) | ART-FS recall | | | | 0.30  (0.21, 0.38) | 0.49  (0.41, 0.57) | 0.81  (0.78, 0.85) |
|  | Clinical records |  | + | - |  |  |  | Clinical records |  | + | - |  |  |  |
|  |  | + | 68 | 64 |  |  |  |  | + | 71 | 73 |  |  |  |
|  |  | - | 88 | 381 |  |  |  |  | - | 85 | 372 |  |  |  |
| ART-FS= AfteR Treatment Follow-up Study; PCOS= polycystic ovarian syndrome  ^a^ Self-report at IVF Study enrollment used as gold standard to calculate specificity and sensitivity  ^b^ Any female factor infertility includes at least one of: PCOS, endometriosis, uterine factor infertility, tubal factor infertility, or diminished reserve/advanced age. | | | | | | | | | | | | | | |
